# Altered Patterns of the Fractional Amplitude of Low-Frequency Fluctuation and Functional Connectivity Between Deficit and Non-Deficit Schizophrenia

**DOI:** 10.3389/fpsyt.2019.00680

**Published:** 2019-09-13

**Authors:** Chao Zhou, Xiaowei Tang, Wei You, Xiang Wang, Xiaobin Zhang, Xiangrong Zhang, Miao Yu

**Affiliations:** ^1^Department of Geriatric Psychiatry, Affiliated Nanjing Brain Hospital, Nanjing Medical University, Nanjing, China; ^2^Department of Psychiatry, Affiliated WuTaiShan Hospital of Medical College of Yangzhou University, Yangzhou, China; ^3^Department of Clinical Medicine, Kangda College of Nanjing Medical University, Lianyungang, China; ^4^Medical Psychological Institute of the Second Xiangya Hospital, Central South University, Changsha, China; ^5^Department of Neurology, Affiliated Nanjing Brain Hospital, Nanjing Medical University, Nanjing, China

**Keywords:** deficit schizophrenia, fractional amplitude of low-frequency fluctuation, salience network, resting-state functional magnetic resonance imaging, neurocognition

## Abstract

**Objective:** A limited number of studies have previously reported on the regional activity [amplitude of low-frequency fluctuation (ALFF)] and functional integration [functional connectivity (FC)] of the whole brain in deficit schizophrenia (DS). The present study investigates the resting-state characteristics of the fractional ALFF (fALFF) and the FC in both DS and non-deficit schizophrenia (NDS) patients, and further explores their correlations with neurocognitive features.

**Methods:** Demographic, resting-state functional magnetic resonance imaging (MRI), and neurocognitive data were collected from 33 DS and 41 NDS male patients, as well as in 40 male healthy controls (HCs). The voxel-wise fALFF was measured to evaluate regional cerebral function. Regions with differences in fALFF between DS and NDS patients were used as seed points in whole-brain FC analysis. Partial correlation analysis was conducted to examine associations between the fALFF or the FC of altered regions and neurocognitive assessments.

**Results:** Both patient groups showed decreased fALFF in the sensorimotor area, visual cortex, and frontoparietal pathway, but increased fALFF in the precuneus and middle cingulate gyrus when compared with the HCs. Moreover, the NDS group demonstrated higher fALFF than HCs in the left thalamus, caudate, and hippocampus. Compared with the NDS group, the fALFF of the visual cortex was specifically increased, but that of the bilateral insula, the anterior cingulate gyrus (ACG), and the regions extended to the frontotemporal cortex was decreased in the DS group. Numerous abnormal FCs of nerve pathways were found between the two patient groups, mainly concentrated in the frontooccipital, frontotemporal, insula-visual cortex, as well as the temporooccipital pathway. Correlation analysis indicated that, in the DS group, the FC value between the left insula and the visual cortex was positively correlated with cognitive flexibility. In the NDS group, the fALFF of the right insula was negatively correlated with speech fluency, and the FC value between the ACG and the visual cortex was positively correlated with visual spatial memory.

**Conclusion:** The present study demonstrates different altered patterns of fALFF and FC between male patients with DS and NDS. The specific altered regions of the salience network (SN) associated with impaired neurocognition in male DS patients suggest novel insights into the pathogenesis of cognitive impairment in schizophrenia.

## Introduction

The spontaneous fluctuations in the low frequency range (0.01–0.08 Hz) of the resting-state blood oxygenation level–dependent (BOLD) signal in the brain are physiologically significant. In 2007, the amplitude of low-frequency fluctuation (ALFF) algorithm was first used in clinical research (1). Since then, studies have successively reported abnormal ALFF in multiple brain regions and neural circuits in patients with schizophrenia, including the medial prefrontal cortex, insula, hippocampus, lingual gyrus, precuneus, and putamen (2–7). Meanwhile, growing evidence indicates that schizophrenia may be the syndrome of a widespread disconnection of neural circuits. With the development of functional magnetic resonance imaging (fMRI), researchers are now able to better explore in depth the neural circuits and intrinsic neural function networks. However, whether cerebral regional activity changes may affect the resting-state functional connection networks such as the central execution network (CEN), the default mode network (DMN), and the salience network (SN) still remains unknown.

Previous studies have reported abnormal changes of functional connectivity (FC) within DMN, CEN, and SN in patients with schizophrenia (8–11). Among these, the SN, because of its mediating effect between functional networks, has attracted widespread attention in recent years (12, 13). The SN, composed of the insula and the anterior cingulate gyrus (ACG), is closely related to the recognition of salient events of internal and external stimuli and is involved in the interaction between brain self-instruction and goal-directed processes (14). The SN plays an important role in transmitting salient signals to other brain networks. Previous fMRI studies have found through the Granger causality test that the insula, the core node in the SN, acts as a switch between the CEN and the DMN (15). It has been speculated that structural and functional abnormalities in the insula, as well as its abnormal regulation of the CEN and DMN, may be involved in the pathogenesis and development of schizophrenia (12, 16). In addition, previous studies have found that abnormalities in the insula may be the basis for impaired coordination of self-monitoring and task performance and may continue to exist as a core feature of schizophrenia (17, 18).

Deficit schizophrenia (DS), distinguished by characteristic primary and persistent negative symptoms, is a clinically homogeneous subgroup of schizophrenia (19–22). DS is considered to differ from non-deficit schizophrenia (NDS) in terms of risk factors, premorbid functioning, disease course, neurobiological correlates, and response to treatment (23–25). Studies on DS better reveal the pathogenesis and development of schizophrenia. However, only a limited number of studies have reported on the whole-brain ALFF and FC of resting-state fMRI in DS patients. One similar study reported increased ALFF in the insula in DS and decreased ALFF in the middle temporal gyrus in NDS compared to healthy controls (HCs) (26), without further performing FC analysis based on ALFF. Furthermore, the correlations between altered ALFF, FC, and neurocognition remain unknown.

The present study measures the differences of fractional amplitude of low-frequency fluctuations (fALFF) between DS, NDS and HCs. In fact, fALFF is normalized ALFF, which may effectively minimize the influence of physiological noise as well as improve sensitivity and specificity in detecting spontaneous brain activities (27). Then we further set the abnormal fALFF regions between DS and NDS groups as seed points that were then used to construct and compare the whole-brain FC network of two patient groups. Furthermore, we explored the correlations between brain imaging findings and cognitive changes. Notably, since male gender had been regarded as one of the risk factors for DS (28, 23), the present study enrolled male patients with chronic schizophrenia in order to increase the homogeneity of participants and to eliminate any potentially confounding findings. We hypothesize that abnormal fALFF exists in brain regions of DS, NDS, and HCs, especially in the insula. In addition, altered FC of the insula is correlated with cognitive assessments in DS.

## Materials and Methods

### Subjects

Seventy-four male schizophrenia patients (33 DS and 41 NDS) were recruited from the psychiatric rehabilitation unit of Yangzhou Wutaishan Hospital, Jiangsu Province, China. The inclusion criteria for the patients are: 1) an explicit diagnosis of schizophrenia according to the *Diagnostic and Statistical Manual of Mental Disorders, Fourth Edition* (*DSM-IV*), and confirmed by the Chinese version of the Structured Clinical Interview for *DSM-IV* (SCID-I) (29); 2) right-handed Chinese Han patients ranging in age from 20 to 65 years; and 3) presenting stable psychiatric symptoms and on antipsychotic medication for at least 12 months before participation. The exclusion criteria of the patients are: a history of previous head trauma, mental retardation, alcoholism or substance abuse, and electroconvulsive therapy. According to the Chinese version of the Schedule for Deficit Syndrome (SDS) (30), patients were divided into two groups: DS and NDS groups. In particular, the SDS was used to assess the deficit syndrome presented in the patients. Patients were classified as DS if they had two of these following symptoms: restricted affect, diminished emotional range, poverty of speech, curbing of interests, diminished sense of purpose, and diminished social drive that are present at a moderately severe level, persistent over 12 months, and are not caused by secondary sources such as medication side effects, depression, paranoia, or anxiety. Forty male HCs, age- and handedness-matched with the patients, were recruited from the local community. Unstructured clinical interviews were conducted to exclude HCs who had a history of organic brain disorders, mental retardation, or severe head trauma as well as a history of personal or family psychiatric disorder. All participants provided written informed consent, which was approved by the Institutional Ethical Committee for clinical research of Zhongda Hospital Affiliated to Southeast University.

### Neurocognitive Assessments

A battery of classical neurocognitive tests was performed for each participant, which comprised the Digit Vigilance Test (DVT), Animal Naming Test (ANT), Controlled Oral Word Association Test (COWAT), Wechsler Adult Intelligence Scale—Chinese Revision (WAIS-RC, block design), Trail Making Test-A, B (TMT-A, B), Stroop Color and Word Test (SCWT), and spatial processing (block design). According to previous studies regarding cognitive processes assessed in schizophrenic patients (31, 32), these cognitive tests were further organized into four rationally motivated cognitive domains: ideation fluency (including ANT and COWAT), cognitive flexibility (including TMT-B and Stroop interference), sustained attention (including DVT, TMT-A, Stroop words, and Stroop colors), and visuospatial memory (including spatial processing test and WAIS-RC). The raw scores of each test were transformed to *Z*-scores according to HCs’ mean and SD, and the composite scores of each cognitive domain were calculated into the mean of the related test *Z*-scores. Cohen’s d effect sizes (33) were computed for each cognitive domain. Particularly, these variables (e.g., TMT) whose values were contrary to the performance were adjusted to their reciprocal before they were transformed to *Z*-scores to ensure the consistency of the statistical direction.

### Data Acquisition

Neuroimaging was conducted using a 3T MR system (GE HDx, Chicago, Illinois) with an eight-channel phased array head coil in the Subei Hospital of Jiangsu Province, Yangzhou, China. Images were acquired using a gradient recalled echo echo-planar imaging (GRE-EPI) sequence with repetition time (TR) = 2,000 ms, echo time (TE) = 25 ms, flip angle = 90°, number of slices = 35, field of view (FOV) = 240 × 240 mm^2^, slice thickness = 4 mm without gap, matrix size = 64 × 64, voxel size = 4 × 4 × 4 mm^3^, and 240 volumes. During the MRI scan, all participants were asked to lie quietly awake in the scanner with eyes closed, and their heads were cozily positioned with cushions inside the coil to minimize head motion. Resting fMRI scanning was sustained for 8 min.

### Preprocessing

The fMRI data were preprocessed using the Statistical Parametric Mapping 8 (SPM8) software (http://www.fil.ion.ucl.ac.uk/spm/software/spm8/) in MATLAB, which was released in 2016 (http://www.mathworks.com/products/matlab/). Briefly, the first 10 images were excluded due to saturation effects and/or magnetization equilibrium. Echo-planar images from each participant were corrected for acquisition time delay between different slices and were realigned to the first volume for head motion correction. The resulting functional images were spatially normalized to the standard space of the Montreal Neurological Institute using an optimum 12-parameter affine transformation and nonlinear deformations (34) and were resampled to 3 mm isotropic voxels. The nuisance signals that included six head motion parameters, cerebrospinal fluid signals, white matter signals, and global mean signals were regressed from the data as corrected values. Finally, the resulting images were smoothed with a 4 mm full width at half maximum Gaussian kernel, after removing the linear trends of time courses. In the present study, we determined the mean framewise displacement (FD) using the Kruskal–Wallis test and there was no significant difference between DS, NDS, and HCs (χ^2^ = 4.307, *P* = 0.116; see **Supplementary Table 1**).

### *f*ALFF Measurement and Functional Connectivity Network Analysis

The Resting-State fMRI Data Analysis Toolkit (REST) (http://resting-fmri.sourceforge.net) was used to perform fALFF measurements and functional connection network analyses. For fALFF measurement, fast Fourier transform was conducted on the time series of whole-brain signal to convert it into a frequency domain power spectrum. Then, root-mean-square calculation was performed on the power spectrum at the range of 0.01–0.08 Hz to obtain the ALFF of the signal. The value of ALFF in this range was added to obtain the total ALFF value, and the fALFF value was obtained by dividing the total value of full-band amplitude from 0.01 to 0.25 Hz. Then, band pass filtering of 0.01–0.08 Hz was carried out to reduce the influence of low-frequency drift and high-frequency physiological noise such as heartbeat and respiratory rhythm. Meanwhile, in order to eliminate the differences of whole-brain fALFF in the overall level between individuals, the fALFF value of each voxel was normalized to the Z value (zfALFF).

In the present study, five abnormal zfALFF regions shared between DS and NDS were used as seed points to construct the FC network map of each seed point to the whole brain. Specifically, the five seed points contained the right fusiform gyrus (FG; coordinates: 42, −51, −18; radius: 6 mm), the left inferior occipital gyrus (IOG; coordinates: −54, −57, −18; radius: 6 mm), the left insula (coordinates: −36, 18, −9; radius: 6 mm), the right insula (coordinates: 36, 12, 3; radius: 6 mm), and the ACG (coordinates: −6, −6, 36; radius: 6 mm) (**Tables 2** and **3**). Pearson correlation analysis was carried out to obtain the correlation coefficient (r value) of mean time series between the seed point and the whole-brain voxel in each participant. Fisher Z transformation was then performed to convert the r value to the Z value, which conforms to the normal distribution. The numerical values resulting from regions of altered fALFF and FC were extracted with REST using altered regions as masks and were later used for correlation analysis.

### Statistical Analysis

Statistical analyses were performed using the Statistical Package for the Social Sciences (SPSS) software version 19.0 (IBM, Armonk, New York). The chi-squared test, the two-sample *t*-tests, and the analysis of variance (ANOVA) were adaptively performed to compare the demographic and neurocognitive variables. The least-significant difference (LSD) was used for *post hoc* comparisons. Analysis of covariance (ANCOVA) was performed using the DPABI (a toolbox for Data Processing & Analysis for Brain Imaging, http://rfmri.org/dpabi) to investigate differences of whole-brain zfALFF and FC among DS, NDS, and HC groups. Age, education, and illness duration were used as covariates. 3dClustSim correction was performed for multiple comparisons at the voxel level (https://afni.nimh.nih.gov/pub/dist/doc/program_help/3dClustSim.html). The statistical threshold was set at a corrected *p* < 0.05. Partial correlation analysis (age, education, chlorpromazine equivalent, and illness duration as covariates) was used to detect the associations between the abnormal zfALFF and the FC of brain regions and neurocognitive performance in both the DS and NDS groups.

## Results

### Demographic and Neurocognitive Assessments

The demographic data collected from all participants are presented in **Table 1**. Significant differences were found for education between groups when analyzed using ANOVA (F_(2,112)_ = 6.685, ANOVA *p* = 0.002); however, no differences were found for age between groups (F_(2,112)_ = 1.464, *p* > 0.05). *Post hoc* comparisons showed that both DS (*p* < 0.05) and NDS (*p* < 0.05) patients had fewer years of education compared to HCs. Regarding the two-sample *t*-tests, except the mean duration of illness (df = 72, *p* = 0.042), no significant differences were found for the age of onset, tobacco usage, and antipsychotic medicine dosage (e.g., chlorpromazine equivalents) between the two patient groups (all df = 72, *p* > 0.05; X_2_ = 1.317, df = 1, *p* = 0.251).

When evaluating neurocognitive characteristics (**Table 1**), *post hoc* comparisons showed that except the DVT in NDS vs. HC (*p* > 0.05), both patient groups performed worse than HCs in all cognitive tests (all *p* < 0.05). DS patients showed worse performance in DVT, TMT-A, TMT-B, Stroop color-only test, Stroop word-only test, COWAT, ANT, and WAIS-RC (block design) (all *p* < 0.05).

**Table 1:** Demographic and cognitive data of DS, NDS, and HC groups.

Variable	DS (n = 33)	NDS (n = 41)	HC (n = 40)	*F*/χ^2^/t/*ES*	P
Age (years)	48.70 ± 7.68	46.02 ± 5.35	45.95 ± 9.47	1.464	0.236
Education (years)	8.82 ± 2.02*	9.20 ± 1.83*	10.60 ± 2.73	6.685	0.002
Age at onset (years)	22.09 ± 3.00	22.51 ± 2.62		−0.644	0.521
Duration of illness (years)	26.61 ± 7.06	23.51 ± 5.81		2.068	0.042^#^
Smoker/non-smoker	20/13	30/11		1.317	0.251^a^
CPZ-equivalent daily dose (mg/day)	503.18 ± 224.38	537.07 ± 209.66		−0.670	0.505
**Sustained Attention**	−10.51 ± 6.47	−4.04 ± 3.27		1.262^b^	
Digit Vigilance Test (s)	292.23 ± 128.61*^,#^	181.74 ± 64.84	137.89 ± 42.86	25.001	<.001
TMT-A (s)	134.21 ± 68.57*^,#^	81.02 ± 30.80*	49.92 ± 23.40	27.238	<.001
Stroop words only	44.39 ± 19.99*^,#^	58.88 ± 15.14*	79.93 ± 16.3l	31.728	<.001
Stroop colors only	26.52 ± 12.85*^,#^	36.05 ± 11.27*	49.87 ± 13.14	24.428	<.001
**Ideation Fluency**	−3.53 ± 1.79	−2.16 ± 2.12		0.698^b^	
Animal Naming Test	9.27 ± 3.22*^,#^	12.07 ± 4.50*	18.10 ± 4.72	33.546	<.001
COWAT	4.91 ± 3.11*^,#^	6.66 ± 3.51*	9.23 ± 2.33	14.300	<.001
**Cognitive Flexibility**	−4.17 ± 2.55	−2.04 ± 1.36		1.042^b^	
TMT-B (s)	302.45 ± 120.19*^,#^	197.90 ± 53.33*	122.64 ± 66.13	34.786	<.001
Stroop interference	16.73 ± 10.69*	21.46 ± 8.56*	32.90 ± 10.47	20.329	<.001
**Visuospatial Memory**	−3.70 ± 2.00	−2.15 ± 1.39		0.900^b^	
WAIS-RC (block design)	13.42 ± 8.92*	21.41 ± 6.45*	27.83 ± 8.31	22.925	<.001
Spatial processing (block design)	11.36 ± 4.47*^,#^	13.29 ± 3.30*	18.25 ± 3.40	25.241	<.001

Data presented as the mean ± SD. DS, deficit schizophrenia; NDS, non-deficit schizophrenia; HC, healthy control; TMT-A, Trail Making Test-A; TMT-B, Trail Making Test-B; COWAT, Controlled Oral Word Association Test; WAIS-RC, Wechsler Adult Intelligence Scale—Chinese Revision; ES, effect size; CPZ, chlorpromazine. The least-significant difference (LSD) was used for post hoc comparisons. The patients’ composite scores of the four neuropsychological domains were standardized using the HC group data. ^a^The P value was obtained by using a chi-squared test. ^b^Effect size (Cohen’s d). *indicates the comparison between patients and HC. *p < 0.05. ^#^indicates the comparison between patients. ^#^p < 0.05.

### z*f*ALFF

**Figure 1:**
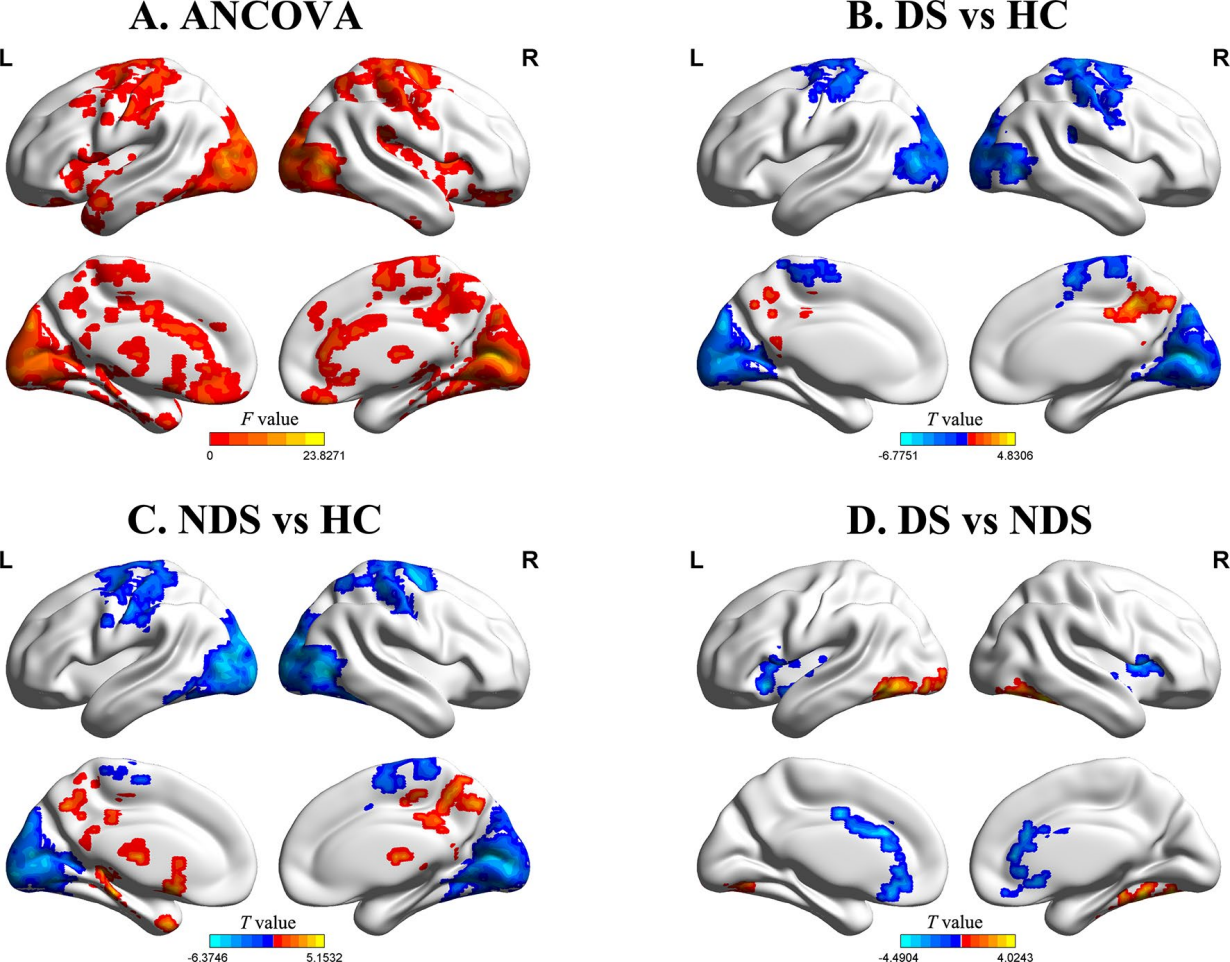
**(A)** Group comparisons of the fractional amplitude of low-frequency fluctuation value of each voxel normalized to the Z value (zfALFF) between deficit schizophrenia (DS), non-deficit schizophrenia (NDS), and healthy control (HC) patient groups. Red represents significantly different regions (voxel level *p* < 0.05, cluster size ≥ 232, corrected *p* < 0.05). **(B**, **C)** Group analyses of the fALFF between patients and HC groups (voxel level *p* < 0.05, cluster size ≥ 426/521, corrected *p* < 0.05). Red and blue respectively represent regions where patients had higher and lower activity compared to HC. **(D)** Group analyses of the zfALFF between DS and NDS groups (voxel level *p* < 0.05, cluster size ≥ 146, corrected *p* < 0.05). Red and blue respectively represent regions where DS showed higher and lower activity compared to NDS.

The results of zfALFF comparisons among DS, NDS, and HCs are presented in **Figure 1** and **Table 2**. Compared to the HCs, both patient groups showed decreased zfALFF in the sensorimotor area, visual cortex, and frontoparietal pathway, as well as increased zfALFF in the precuneus and the middle cingulate gyrus. In addition, compared to the HCs, the NDS group also showed increased zfALFF in the left thalamus, the caudate nucleus, and the hippocampus (**Figures 1A−C**). Compared with the NDS group, the zfALFF of the visual cortex was specifically increased, but decreased in regions such as the bilateral insula, the ACG/ventromedial prefrontal cortex (VMPFC), and related regions extended to the frontotemporal cortex in the DS group (**Figure 1D**). In the NDS group, the zfALFF value of the right insula was negatively correlated with speech fluency (r = −0.353, *p* = 0.032, uncorrected). Additionally, in the DS group, no correlation was found between altered zfALFF and neurocognitive function.

**Table 2:** Altered regions of zfALFF among DS, NDS, and HCs.

Regions	Hemisphere	Peak MNI coordinates			*F*/*T* score	Cluster size
		x	y	z		
**ANCOVA**						
Cuneus/middle occipital gyrus/middle frontal gyrus/lingual gyrus/middle temporal gyrus/insula	Bilateral	45	−69	0	23.827	9,150
Insula/superior temporal gyrus/middle temporal gyrus/inferior frontal gyrus	Left	−51	−12	12	11.382	404
**DS vs. HC**						
Cuneus/middle occipital gyrus/lingual gyrus/middle temporal gyrus	Bilateral	12	−81	9	−6.775	2,830
Pre/postcentral gyrus/middle frontal gyrus/inferior parietal lobule/medial frontal lobe	Bilateral	12	−27	72	−5.642	1,417
Precuneus/middle cingulate gyrus	Bilateral	0	−51	42	4.831	500
**NDS vs. HC**						
Cuneus/middle occipital gyrus/inferior occipital gyrus/lingual gyrus/calcarine gyrus/middle temporal gyrus	Bilateral	−24	−96	−3	−6.375	3,592
Pre/postcentral gyrus/middle frontal gyrus/superior frontal gyrus/inferior parietal lobule	Left	−27	3	51	−5.445	762
Pre/postcentral gyrus/superior frontal gyrus/middle frontal gyrus	Right	27	−9	63	−5.892	812
Thalamus/caudate nucleus/hippocampus/parahippocampal gyrus	Left	−3	15	6	4.693	701
Precuneus/middle cingulate gyrus	Bilateral	0	−57	45	5.153	627
**DS vs. NDS**						
Fusiform gyrus/inferior temporal gyrus	Right	42	−51	−18	4.024	239
Inferior occipital gyrus/middle occipital gyrus/fusiform gyrus/inferior temporal gyrus	Left	−54	−57	−18	3.968	210
Insula/inferior frontal gyrus/superior temporal gyrus	Left	−36	18	−9	−4.455	175
Insula/superior temporal gyrus	Right	36	12	3	−4.463	154
Anterior cingulate gyrus/ventromedial prefrontal cortex	Bilateral	−6	−6	36	−4.490	276

zfALFF, fractional amplitude of low-frequency fluctuation value of each voxel normalized to the Z value; MNI, Montreal Neurological Institute; ANCOVA, analysis of covariance.

### Functional Connectivity Network

**Figure 2:**
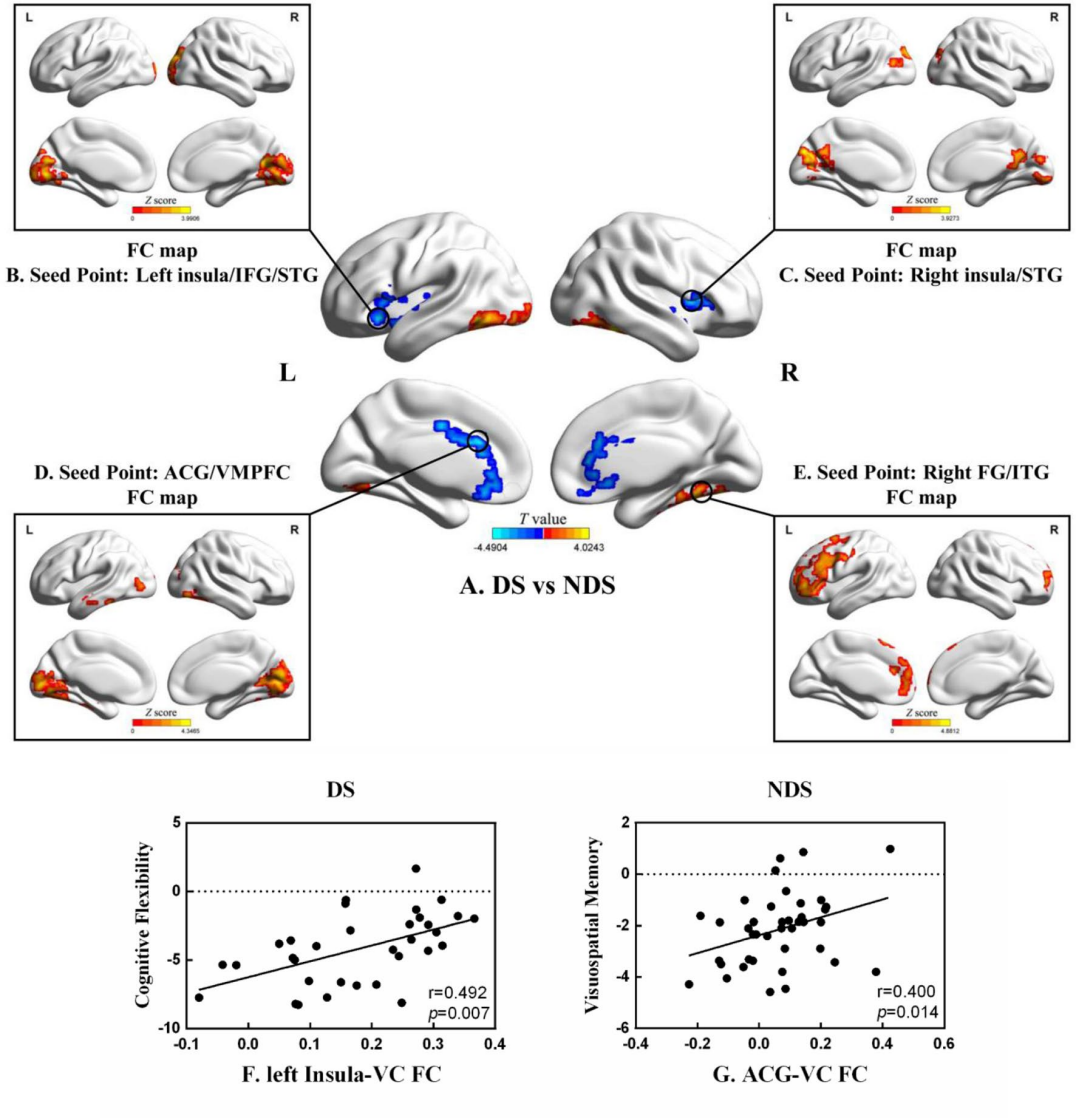
**(A)** Group analyses of the zfALFF between DS and NDS groups. **(B**–**E)** Functional connectivity analysis based on five seed points between DS and NDS groups. (There was no significantly different region contained in one seed point FC map.) Red represents regions where DS had higher FC compared to NDS. FC, functional connectivity; IFG, inferior frontal gyrus; STG, superior temporal gyrus; ACG, anterior cingulate gyrus; VMPFC, ventromedial prefrontal cortex; FG, fusiform gyrus; ITG, inferior temporal gyrus. The statistical threshold was set with a combination of voxel level *p* < 0.05 and cluster size ≥ 465/465/449/464 voxels for four ROI FC analyses (DS vs. NDS), which corresponds to a corrected *p* < 0.05. **(F**, **G)** Correlations between the FC of altered regions and neurocognitive assessments in both DS and NDS patients. VC, visual cortex. The significance threshold was set at *p* < 0.05.

As shown in **Table 3** and **Figure 2**, numerous abnormal FCs of nerve pathways were found between the two patient groups, mainly concentrated in the frontooccipital, frontotemporal, and insula-visual cortex regions, as well as the temporooccipital pathway. Compared to the NDS group, all the FC values of the brain regions mentioned above were significantly greater in the DS group. In addition, in the DS group, the FC value between the left insula and visual cortex was positively correlated with cognitive flexibility (r = 0.492, *p* = 0.007, uncorrected, **Figure 2F**). In the NDS group, the FC value between the ACG and the visual cortex was positively correlated with visual spatial memory (r = 0.400, *p* = 0.014, uncorrected, **Figure 2G**).

**Table 3:** Altered regions of FC based on the ROI analysis between DS and NDS.

ROI Seed Points	Regions (DS vs. NDS)	Hemisphere	Peak MNI coordinates			*Z*-score	Cluster size
			x	y	z		
Right FG/ITG	Superior frontal gyrus/middle frontal gyrus/inferior frontal gyrus/ventromedial frontal cortex	Left	−39	45	0	4.881	1,676
Left IOG/MOG/FG/ITG	None						
Left insula/IFG/STG	Cuneus/lingual gyrus/calcarine gyrus/superior occipital gyrus	Bilateral	21	−72	−3	3.991	1,905
Right insula/STG	Cuneus/calcarine gyrus/middle occipital gyrus/middle temporal gyrus	Bilateral	−6	−81	30	3.927	1,015
ACG/VMPFC	Calcarine gyrus/lingual gyrus/fusiform gyrus/left inferior temporal gyrus	Bilateral	−15	−78	−3	4.347	1,860

MNI, Montreal Neurological Institute; FG, fusiform gyrus; ITG, inferior temporal gyrus; IOG, inferior occipital gyrus; MOG, middle occipital gyrus; IFG, inferior frontal gyrus; STG, superior temporal gyrus; ROI, region of interest; ACG, anterior cingulate gyrus; VMPFC, ventromedial prefrontal cortex.

## Discussion

The present study demonstrates the abnormal fALFF and FC between DS, NDS, and HCs, as well as the associations between resting brain function activities and neurocognition in both the DS and NDS groups. Specifically, the main findings include 1) decreased fALFF of the left insula and frontotemporal cortex in the DS group compared to the NDS and HCs groups; 2) increased fALFF of the bilateral insula and ACG, and decreased fALFF of the bilateral visual cortex in the NDS group compared to the DS and HCs groups; 3) greater FC of the frontooccipital, frontotemporal, and insula-visual cortex regions, as well as the temporooccipital pathway in the DS group, compared to the NDS group; and 4) the association between the FC of the left insula and the visual cortex and cognitive flexibility in the DS group, as well as the association between the FC of the ACG and the visual cortex and visual memory in the NDS group. To the best of our knowledge, this is the first study that demonstrates the altered patterns of fALFF and relevant neurocognitive associations in patients with DS and NDS.

Previous studies on the abnormal functional changes of the insula in patients with schizophrenia are inconsistent. Increased ALFF and decreased fALFF were both reported in the insula of schizophrenia patients (7, 35). The inconsistency of these results may be partly due to the use of different segmentation methods of brain structures (36) and functional imaging indexes (35, 37). However, the more important reason may be the heterogeneity of schizophrenia itself. In the present study, we demonstrated the decreased fALFF of the left insula in the DS group. A previous large meta-analysis reported a thinner cortex of the bilateral insula in schizophrenia when compared to healthy volunteers, and further suggested correlations between earlier age of onset and longer duration of illness and thinner right insula cortical thickness (38). Previous morphological studies of DS also reported a decreased volume of the left insular gray matter in chronic (36, 39) and first-episode drug-naïve (40) DS patients, both compared to NDS and HC groups. Furthermore, previous studies suggest that the decreased volume of insular gray matter in DS patients is positively correlated with the severity of negative symptoms (40).

It is worth noting that in the current study, the fALFF of the bilateral insula was specifically increased in the NDS group. The lateralization and adverse changes of fALFF in the insula between the DS and NDS groups may partially account for the inconsistent results in previous schizophrenia studies. The fALFF patterns vary substantially among different studies where DS and NDS patients were recruited at different proportions. The distinct altered patterns of fALFF in the present study may be due to primary/secondary negative symptoms in DS/NDS patients. Abnormal insular ALFF has been previously found to correlate with mental symptoms. Increased ALFF in the insula is reported to be associated with anxiety [Stein et al., (41)], while decreased ALFF is associated with autism (42).

The ACG as one of the subregions of prefrontal cortex would be active when cognitive control is required (43, 44). A previous study regarding an emotional picture-rating task reported that schizophrenia patients with high negative symptoms had reduced ACG activation in response to pleasant images relative to HCs, demonstrating the functional significance of the relationship between negative symptoms and ACG dysfunction in schizophrenia (45). In the present study, the decreased ACG zfALFF in DS compared to NDS may be related to the characteristic primary and persistent negative symptoms of DS. Furthermore, increased ACG ALFF was found in individuals with genetic high risk for schizophrenia compared to HCs (46), suggesting that the increased zfALFF in NDS may emerge and exist before the onset of the disease. The other regions with differences between DS and NDS, such as the visual cortex, further demonstrate the differently altered patterns of fALFF in the subgroups of schizophrenia.

In the present study, differences of FC in the frontooccipital, frontotemporal, and insula-visual cortex regions, as well as the temporooccipital pathway, were found between the two patient groups. It is worthwhile to note that, compared to the NDS group, all the FCs of the mentioned areas were increased in the DS group. Specifically, the fALFF of the left insula, the right insula, and the ACG decreased, while the FCs of these regions increased in the DS group, both as compared to the NDS group. Previous studies on multi-regions and multi-network functional connections (FCs) found that the FCs between the insula/ACG and other brain regions, such as the temporal lobe, the corpus striatum, the prefrontal lobe, and the supplementary motor area, were significantly decreased in schizophrenia patients as compared to HCs (18, 47). One possible explanation is that the increased FC of these regions in DS may be a compensation for the altered activity in the region. Furthermore, the abnormal increased FCs in DS compared to NDS suggests that DS might be a specific subgroup within schizophrenia.

The present study demonstrates the correlation between the FC of the left insula and visual cortex and cognitive flexibility in the DS group, and the correlation between the FC of the ACG and visual cortex and visual spatial memory in the NDS group. The different association patterns between resting brain functional signals and neurocognition in DS and NDS groups may provide a new perspective for understanding the pathogenesis of cognitive impairments in schizophrenia. In addition, previous studies on the FC of the insula in patients with schizophrenia have demonstrated the abnormal FC between the insula and the higher-order visual areas, suggesting an association with the impaired cognitive function of recognizing facial emotional expressions in schizophrenia (17). Usually, the generation of higher-order visual signals needs to be completed through the inferior temporal gyrus–inferior longitudinal fasciculus–visual cortex and relay processing by the insula. The abnormal FC between the insula and the visual cortex within the two patient groups may further suggest altered function of facial emotion recognition and social communication in DS patients.

In the present study, some limitations should be discussed. First, all the schizophrenia patients were treated with antipsychotics, which may still have the potential to affect brain FC and neural activity by antagonizing dopamine or other possible receptors (48, 49). However, the present study suitably matched the types and dosages of antipsychotics used in the two patient groups, and the dose of antipsychotics was not significantly correlated with any resting brain function signal. Secondly, the patients enrolled in this study were all male inpatients with chronic schizophrenia. Although the purpose was to increase the homogeneity of participants, future studies should collect larger data sets that include female subjects to consider important gender differences and to improve the statistical power of the study. Finally, this study should be considered as an exploratory analysis where the *p* < 0.05 (after correction) level was used for statistical validity. Subsequent studies could further expand the sample size and perform stricter statistical correction to explore essential changes in the structure or function of the brain in DS and NDS patients.

In summary, the present study demonstrated different altered patterns of the fALFF and of FC within DS and NDS patient groups. DS is mainly manifested as decreased neural activity in the left insula, and NDS is manifested as increased neural activity in the bilateral insula. The specific altered region associated with impaired neurocognition in DS patients can potentially be used as a biomarker and a treatment target for schizophrenia.

## Ethics Statement

The research was approved by the Institutional Ethical Committee for clinical research of ZhongDa Hospital affiliated to Southeast University and all participants provided informed consent (2013ZDSYLL52.0).

## Author Contributions

XRZ and XBZ designed and organized the research. XW provided the Schedule for Deficit Syndrome (SDS). XT and MY collected the imaging and cognitive data. CZ, WY, and MY analyzed the data and wrote the manuscript.

## Funding

This work was supported by the National Key Research and Development Program of China (2018YFC1314303 and 2016YFC1307000), the National Natural Science Foundation of China (NSFC) (nos. 31671144, 81701675 and 81371474), and the medical key talent projects in Jiangsu Province (ZDRCA2016075).

## Conflicts of Interest Statement

The authors declare that the research was conducted in the absence of any commercial or financial relationships that could be construed as a potential conflict of interest.

## Abbreviations

DS, deficit schizophrenia; NDS, non-deficit schizophrenia; SN, salience network; CEN, central execution network; DMN, default mode network; BOLD, blood oxygenation level–dependent; ALFF, amplitude of low-frequency fluctuation; *DSM-IV*, *Diagnostic and Statistical Manual of Mental Disorders, Fourth Edition*; SCID-I, Chinese version of the Structured Clinical Interview for *DSM-IV*; SDS, Schedule for Deficit Syndrome; FC, functional connectivity; IFG, inferior frontal gyrus; STG, superior temporal gyrus; ACG, anterior cingulate gyrus; VMPFC, ventromedial prefrontal cortex; FG, fusiform gyrus; ITG, inferior temporal gyrus; VC, visual cortex; TMT, Trail Making Test; COWAT, Controlled Oral Word Association Test; WAIS-RC, Wechsler Adult Intelligence Scale—Chinese Revision.
