# Supplementary material for: Altered Patterns of the Fractional Amplitude of Low-Frequency Fluctuation and Functional Connectivity Between Deficit and Non-Deficit Schizophrenia
Source: Front Psychiatry. 2019 Sep 13;10:680. doi: 10.3389/fpsyt.2019.00680 (PMC6754073; doi:10.3389/fpsyt.2019.00680)
Supplement: Supplementary file 1 [file Table_1.docx]

**Supplementary Table 1**

Clinical and head motion features between DS, NDS and HCs groups

|  | DS | NDS | HCs | *t*/χ^2^ | *P* |
| --- | --- | --- | --- | --- | --- |
| **BPRS Total** | 32.15±3.05 | 27.41±2.42 |  | 7.450 | <0.001^#^ |
| Positive syndrome | 6.09±1.07 | 6.34±1.09 |  | -0.992 | 0.324 |
| Negative syndrome | 12.58±1.70 | 7.46±0.95 |  | 15.467 | <0.001^#^ |
| Disorganized syndrome | 6.61±1.35 | 6.46±0.81 |  | 0.564 | 0.574 |
| Affect | 6.88±1.17 | 7.15±1.15 |  | -0.988 | 0.327 |
| **SANS Total** | 57.36±8.46 | 32.37±5.86 |  | 14.426 | <0.001^#^ |
| Affective flattening | 3.06±0.66 | 1.83±0.50 |  | 9.180 | <0.001^#^ |
| Alogia | 2.91±0.72 | 1.51±0.51 |  | 9.759 | <0.001^#^ |
| Avolition-apathy | 2.91±0.68 | 1.68±0.52 |  | 8.792 | <0.001^#^ |
| Anhedonia-asociality | 2.97±0.81 | 1.54±0.64 |  | 8.529 | <0.001^#^ |
| Attention | 2.64±1.03 | 1.68±0.82 |  | 4.447 | <0.001^#^ |
| **SAPS Total** | 8.85±3.71 | 9.85±4.32 |  | -1.058 | 0.293 |
| Hallucinations | 0.45±0.51 | 0.54±0.81 |  | -0.508 | 0.613 |
| Delusions | 1.06±0.35 | 1.17±0.50 |  | -1.121 | 0.266 |
| Bizarre behavior | 0.82±0.53 | 1.00±0.59 |  | -1.378 | 0.172 |
| Positive thought disorder | 1.03±0.81 | 1.00±0.63 |  | 0.176 | 0.861 |
| **SDS total score** | 11.21±2.61 | 4.32±2.34 |  | 11.976 | <0.001^#^ |
| Avolition | 6.06±1.52 | 2.66±1.46 |  | 9.786 | <0.001^#^ |
| Poor emotional expression | 5.15±1.33 | 1.66±1.11 |  | 12.341 | <0.001^#^ |
| Head Motion (mean FD) | 0.17±0.08 | 0.20±0.14 | 0.15±0.07 | 4.307^a^ | 0.116 |

Note: ^#^ indicates significant differences between DS and NDS, *p* <0.05. ^a^ indicates Kruskal-Wallis Test, χ^2^. BPRS, Brief Psychiatric Rating Scale; SANS, the Scale for the Assessment of Negative Symptoms; SAPS, the Scale for the Assessment of Positive Symptoms; FD, Framewise Displacement.
